# A Portable Support Attitude Sensing System for Accurate Attitude Estimation of Hydraulic Support Based on Unscented Kalman Filter

**DOI:** 10.3390/s20195459

**Published:** 2020-09-23

**Authors:** Xuliang Lu, Zhongbin Wang, Chao Tan, Haifeng Yan, Lei Si, Dong Wei

**Affiliations:** School of Mechatronic Engineering, China University of Mining and Technology, Daxue Road, Xuzhou 221116, China; LuXuliang@cumt.edu.cn (X.L.); tccadcumt@126.com (C.T.); YanHaifeng@cumt.edu.cn (H.Y.); lei.si@cumt.edu.cn (L.S.); weidongcmee@cumt.edu.cn (D.W.)

**Keywords:** support attitude, inertial measurement unit, coal mining, unscented Kalman filter, quaternion, gradient descent

## Abstract

To measure the support attitude of hydraulic support, a support attitude sensing system composed of an inertial measurement unit with microelectromechanical system (MEMS) was designed in this study. Yaw angle estimation with magnetometers is disturbed by the perturbed magnetic field generated by coal rock structure and high-power equipment of shearer in automatic coal mining working face. Roll and pitch angles are estimated using the MEMS gyroscope and accelerometer, and the accuracy is not reliable with time. In order to eliminate the measurement error of the sensors and obtain the high-accuracy attitude estimation of the system, an unscented Kalman filter based on quaternion according to the characteristics of complementation of the magnetometer, accelerometer and gyroscope is applied to optimize the solution of sensor data. Then the gradient descent algorithm is used to optimize the key parameter of unscented Kalman filter, namely process noise covariance, to improve the accuracy of attitude calculation. Finally, an experiment and industrial application show that the average measurement error of yaw angle is less than 2° and that of pitch angle and roll angle is less than 1°, which proves the efficiency and feasibility of the proposed system and method.

## 1. Introduction

Hydraulic support is an important safety support equipment in the automatic coal mining working face, which is of great significance to the safety of coal miners and the normal operation of coal mining equipment. In recent years, with the continuous development of intelligent mining technology [1,2], the roboticized hydraulic support technology has been paid more and more attention by scholars and coal mining managers. In particular, the precise estimation of the support attitude of hydraulic support directly determines the intelligent ability of hydraulic support. Therefore, it is urgent to study a new attitude sensing method to obtain accurate support attitude of hydraulic support [3], especially in the special application scenarios with a large demand for the number of sensors and complex environment.

The existing studies on the support attitude sensing measurement of hydraulic support are mainly focused on the mechanism and kinematics of hydraulic support and trying to achieve the accurate estimation of support attitude by kinematic analysis of its mechanism [4]. In [5,6], Polish scholars analyzed the structural composition and empirical formula of support strength of hydraulic support, and developed a new type of support mechanism for vertical displacement of hydraulic support, but which can only approximately estimate the yaw angle of hydraulic support by experience. In [7,8], through the analysis of the four-linkage mechanism and parameters of hydraulic support, the kinematics equation is established, and then the support attitude can only be roughly estimated due to the neglected clearance between the hinge joint and the pin shaft. In [9], a laser radar detection method is used for measuring the relative attitude angle of hydraulic support based on the position of inspection robot, but the estimation result is disturbed greatly due to the influence of robot motion vibration. In [10,11], a mechanical-electrical-hydraulic coordination technique based on the dynamic response of the load is exploited to adjust the attitude of hydraulic support, but it is easily affected by the pressure of the pump station. In [12], a virtual adjustment method of support attitude under the propulsive state of hydraulic support groups is proposed and the estimation accuracy is highly dependent on the modeling precision of the virtual model, and different hydraulic supports need different virtual models, which is difficult to establish and has poor universality. In [13], an approach for monitoring the posture of hydraulic support with fiber Bragg grating tilt sensor based on gravity is presented, but it is not applied to measure yaw angle of the canopy. However, in automatic coal mining working face with the complicated geological structures, the above methods are characterized by complex solutions, difficult modelling, large estimation disturbance and lack of universality with estimating attitude or relative attitude indirectly based on kinematic parameters of mechanism, or can only estimate a single attitude angle. In this paper, we try to explore a new approach for estimating directly the support attitude of hydraulic support with comprehensive consideration of estimation accuracy, measurement method, universality and sensor size.

Inertial measurement unit (IMU) can be independent of the existing mechanism of hydraulic support to estimate support attitude directly, which is not affected by the mechanism of hydraulic support itself. In recent years, the development of microelectromechanical system technology has accelerated leaps and bounds the development process of IMU, which makes the IMUs become low in cost, low in power consumption and small in size. Additionally, it is widely applied in robotics [14,15], navigation [16,17] and virtual reality [18]. Scientific research is not limited to sensor applications [19], but also includes the performance of IMUs [20]. The performance improvement and calibration analysis of IMU provide precise attitude estimation technologies for its application [21], such as the method of improving estimation performance based of sine rotation vector; the acceleration and magnetic field measurements are transformed into the differences between the Euler angles of the measured attitude and the predicted attitude to correct the predicted attitude [22]. However, the measurement accuracy and performance of these low-cost MEMS inertial measurement units are easily limited by the complex working environment [23,24], especially the automatic coal mining working face with complex magnetic interference produced by coal structure and high-power electromechanical equipment. Therefore, it is necessary to use a filtering algorithm to improve the estimation accuracy.

The Kalman filter (KF) and particle filter (PF) are the most studied and widely used filtering algorithms. The Kalman filter for linear filtering and the extended Kalman filter (EKF) with Jacobian matrix and the unscented Kalman filter (UKF) for nonlinear filtering are frequently used to process inertial information, including object tracking [25] and rotation estimation [26]. In order to reduce yaw drift of attitude rotation estimation in indoor scenarios, an improved EKF combining INS mechanization algorithm and zero velocity update methodology is proposed to improve the accuracy of yaw estimation [27], but the Jacobian matrix is used to update the state transition matrix and the observation matrix in the linearization steps of system equation in attitude determination, which leads to poor stability and large estimation bias. Owing to the system, equations are updated based on the sigma-point approximation generated by unscented transformation in UKF [28,29], which has been demonstrated to have more attitude solution accuracy and strong robustness than EKF in estimating attitude with highly nonlinear kinematics models. In [30,31], a quaternion-based MEMS sensors fusion algorithm and heading estimation approach based on rotation matrix and principal component analysis are proposed to improve the heading estimation accuracy of indoor pedestrians, which shows that quaternion is easier to implement than other methods in sensor fusion technology. However, the particle filter is a sequential importance sampling filtering method based on Bayesian estimation [32] and its idea is based on the Monte Carlo method to represent probability with particle set, which can be used in any state space models [33,34]. In [35], a method of filtering and predicting time-varying signals under model uncertainty is proposed to realize dynamic estimation of the data. In [36], a particle metropolis Hastings algorithm driven by multiple parallel particle filters is proposed to make inference on dynamic and static variables. In any case, although the particle filter has good estimation performance in nonlinear filtering, the mathematical process of particle filters is more complex, and the algorithm implementation and calculation are difficult. Moreover, the resampling, particle degradation and calculation amount in the calculation process lead to the complexity of the implementation process and poor real-time performance. Therefore, the operation time is much longer than that of UKF.

Through the above research on attitude estimation of nonlinear systems, and considering the estimation accuracy, calculation speed and real-time performance of algorithm, UKF is proved to be a powerful technique for estimating attitude angle and a superior alternative to the EKF and PF in various nonlinear system filtering [37,38]. Unfortunately, the key parameter of UKF selected by experience, process noise covariance Q, has a great influence on the estimation accuracy in the complex working environment [39], so the research on tuning of parameter Q is very meaningful [40,41]. Therefore, the gradient descent algorithm [42] with the advantages of fast global convergence, faster operation speed and simple realization could be used to tune the process noise covariance Q to improve the estimation performance of UKF.

In this paper, a support attitude sensing system with a character of intrinsic safety is designed to measure the support attitude of hydraulic support in the special application scenarios with a large demand for the number of sensors and complex environment. The designed support attitude sensing system is a nine-axis low-cost MEMS measurement unit composed of a gyroscope, magnetometer and accelerometer. The proposed gradient descent algorithm-optimized quaternion-based unscented Kalman filter makes full use of the characteristics of complementation of the magnetometers without long-term drift, accelerometers and gyroscopes which are not affected by magnetic disturbance to improve the support attitude estimation accuracy of hydraulic support.

The remaining parts of this paper are organized as follows. In Section 2, the support attitude sensing system designed in our laboratory is described. Section 3 introduces the proposed optimized quaternion-based unscented Kalman filter based on the complementary characteristics of MEMS sensors in detail. In Section 4, an experiment is conducted and analyzed to validate the estimation performance of the proposed system and approach. Then, industrial applications and tests are carried out in Section 5. Section 6 summarizes our conclusions and future work.

## 2. Support Attitude Sensing System

### 2.1. Establishing the Coordinate System and Attitude Angle

**Figure 1:**
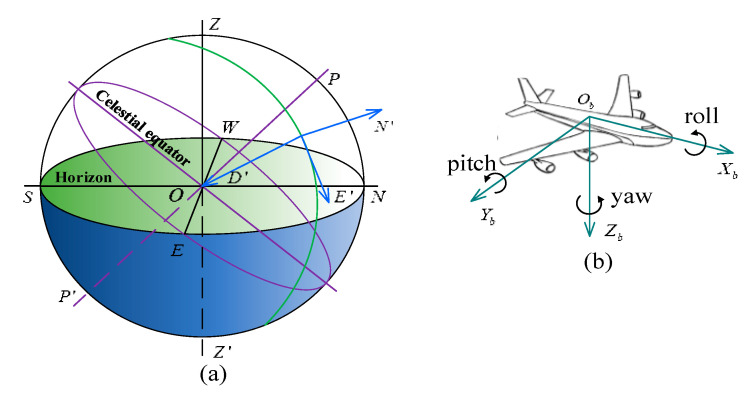
Definition of coordinate systems and carrier attitude angles: (**a**) local geographical coordinate system (*N’E’D’*) defined relative to the geoid; (**b**) carrier coordinate system (*X_b_Y_b_Z_b_*) and carrier attitude angles including roll, pitch and yaw angles.

The two coordinate systems and three attitude angles need to be established in the support attitude measurement of hydraulic support, as shown in Figure 1, which are the geographical coordinate system, carrier coordinate system, pitch angle, yaw angle and roll angle. The geographical coordinate system (frame n) is the North-East-Down (NED) orthogonal coordinate defined by the relative geoid. The Down axis (D axis) is perpendicular to the ellipsoid of the reference system and points to the interior of the earth. The North axis (N axis) points to true north (the velocity vector projection along the earth’s rotation angle on the plane perpendicular to the D axis). Finally, the East axis (E axis) points horizontally east and completes the right-hand orthogonal coordinate system. The carrier coordinate system (frame b) is fixed to the carrier, which is the reference coordinate system. The $Z_{b}$ axis and $X_{b}$ axis point to the bottom and forward along the longitudinal axis of the carrier, respectively. The $Y_{b}$ axis points to the right wing (side) and completes the right-hand orthogonal coordinate system with the axes $X_{b}$ and $Z_{b}$.

The support attitude of hydraulic support includes yaw angle, roll angle and pitch angle. The yaw angle of rotation around $Z_{b}$ axis of the carrier is the angle between the N axis and the longitudinal axis $X_{b}$ of the carrier, measured on the horizontal plane and clockwise is positive. The pitch angle of rotation around the $Y_{b}$ axis of the carrier is the angle between the horizontal plane and the longitudinal axis $X_{b}$ of carrier, measured on the vertical plane and the direction of the carrier’s head up is positive. The roll angle of rotation around the $X_{b}$ axis of the carrier is the angle between the horizontal plane and the cross axis $Y_{b}$ of the carrier, measured on the cross section, and the lifting direction of the carrier on the left side is positive.

**Figure 2:**
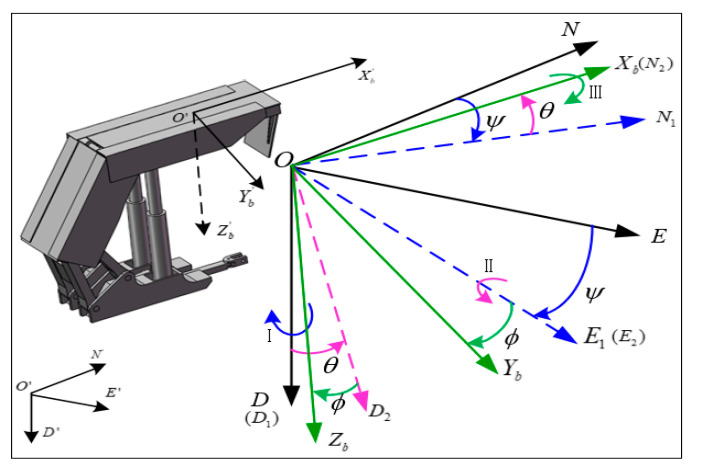
Coordinate transformation and directional cosine matrix definition of the canopy between frame b and frame n based on hydraulic support.

The carrier coordinate system and NED coordinate system can coordinate transformation with each other by rotating yaw angle, pitch angle and roll angle around the X axis, Y axis and Z axis in turn, and the transformation relationship from ONED to $OX_{b}Y_{b}Z_{b}$ is shown in Figure 2. The coordinate transformation matrix is also called direction cosine matrix, which is written as:

(1) $$\begin{array}{l} C_{n}^{b}={\left[\phi \right]}_{N_{2}}{\left[\theta \right]}_{E_{1}}{\left[\psi \right]}_{D}\\ =\left[\begin{array}{ccc} \cos\theta \cos\psi & \cos\theta \sin\psi & -\sin\theta \\ \cos\psi \sin\phi \sin\theta -\cos\phi \sin\psi & \sin\phi \sin\theta \sin\psi +\cos\phi \cos\psi & \sin\phi \cos\theta \\ \cos\phi \sin\theta \cos\psi +\sin\phi \sin\psi & \cos\phi \sin\theta \sin\psi -\sin\phi \cos\psi & \cos\phi \cos\theta \end{array}\right] \end{array}$$

On the contrary, the transformation from $OX_{b}Y_{b}Z_{b}$ to ONED is written as:

(2) $$C_{b}^{n}=\left[\begin{array}{ccc} \cos\theta \cos\psi & \sin\phi \sin\theta \cos\psi -\cos\phi \sin\psi & \cos\phi \sin\theta \cos\psi +\sin\phi \sin\psi \\ \cos\theta \sin\psi & \sin\phi \sin\theta \sin\psi +\cos\phi \cos\psi & \cos\phi \sin\theta \sin\psi -\sin\phi \cos\psi \\ -\sin\theta & \sin\phi \cos\theta & \cos\phi \cos\theta \end{array}\right]$$

where ϕ, θ and ψ are roll angle, pitch angle and yaw angle of support attitude of hydraulic support canopy, respectively.

### 2.2. Intrinsically Safe Micro Inertial Sensor

The support attitude sensing system based on the intrinsically safe micro inertial sensor is designed as a small strapdown inertial navigation system in our lab to meet the coal mine special environment navigation requirements of hydraulic support, mine inspection robot, support robot and driving robot. The intrinsically safe micro inertial sensor combining the IMU and magnetometer is composed of a three-axis gyroscope, three-axis magnetometer, three-axis accelerometer, an intrinsically safe microprocessor and a flameproof shell. The three single-axis gyroscopes and accelerometer use the ICM-42605 with the measuring range of ±125°/s and ±16 g. The three single-axis magnetometers use the LSM3030C with a measurement range of ±16 gauss magnetic full scale. STM32MP157 is selected as the microprocessor of the system and it is based on the high-performance dual-core Arm^®^ Cortex^®^-A7 32-bit RISC core with an operating frequency of up to 650 MHz. The support attitude sensing system uses an I2C/SPI bus for digital output.

### 2.3. Support Attitude Estimation

According to the mining process of automatic coal mining working face, there are two working states in the support process for the canopy of hydraulic support, namely, static working state and moving working state. An accelerometer with superior static characteristics has high measurement accuracy in static state. A gyroscope with stable static performance has high measurement accuracy in moving state. A magnetometer with the measurement stability in a long time test is easily disturbed by the external perturbed magnetic sources produced by coal rock structure and high-power shearer equipment. Nevertheless, the gyroscope is not disturbed by magnetic sources from the automatic coal mining working face. Therefore, this paper proposes a new attitude estimation method for measuring the pitch angle, yaw angle and roll angle of hydraulic support based on the characteristics of complementation of accelerometers, gyroscopes and magnetometers, which eliminates the errors derived from the MEMS sensors by the quaternion-based UKF.

**Figure 3:**
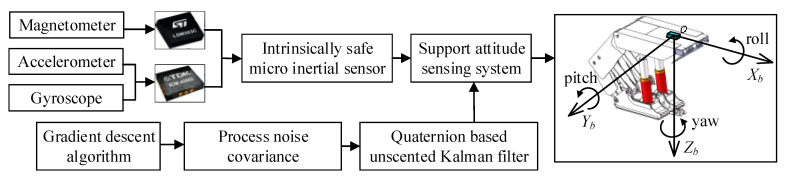
Block diagram of support attitude sensing system based on unscented Kalman filter for attitude measurement according to the complementary characteristics of the MEMS inertial sensor.

In addition, the tuning parameter of UKF, the process noise covariance Q, is usually a general value set by experience, which sometimes makes UKF produce large estimation errors, especially in a complex environment. Therefore, a gradient descent algorithm is used to optimize Q to improve the estimation accuracy and performance in this paper. The flow chart of accurate estimation of support attitude of hydraulic support using the quaternion-based unscented Kalman filter through tuning process noise covariance is shown in Figure 3. The other key tuning parameter, the observation noise covariance R, is related to the deviation of the magnetometer, accelerometer and gyroscope, which can be obtained by calculating the expectation of the square of deviation of the sensors.

#### 2.3.1. Support Attitude Estimation Based on Magnetometer

The magnetometer output is the sensitive geomagnetic field intensity. On the earth’s surface, the geomagnetic field always points to the north along the magnetic induction, with components in the north direction and vertical direction of that, and no component in the east direction. Therefore, magnetic field coordinate is (N, 0, D) in the geomagnetic coordinate system. The magnetometer output is $M_{n}^{mag}={\left[\begin{array}{ccc} M_{N} & 0 & M_{D} \end{array}\right]}^{T}$ when the geomagnetic coordinate system coincides with the frame b. The magnetometer output is $M_{n}={\left[\begin{array}{ccc} M_{x} & M_{y} & M_{z} \end{array}\right]}^{T}$ in frame n and $m_{b}={\left[\begin{array}{ccc} m_{x} & m_{y} & m_{z} \end{array}\right]}^{T}$ in frame b. According to $m_{b}=C_{n}^{b}{|}_{\psi =\psi_{m}}\cdot M_{n}^{mag}$, the relationship of geomagnetic field intensity between the two coordinate systems can be obtained as:

(3) $$\left[\begin{array}{c} m_{x}\\ m_{y}\\ m_{z} \end{array}\right]=\left[\begin{array}{c} M_{N}\cos\theta \cos\psi_{m}-M_{D}\sin\theta \\ M_{D}\sin\phi \cos\theta +M_{N}\left(\sin\phi \sin\theta \cos\psi_{m}-\cos\phi \sin\psi_{m}\right)\\ M_{D}\cos\phi \cos\theta +M_{N}\left(\cos\phi \sin\theta \cos\psi_{m}+\sin\phi \sin\psi_{m}\right) \end{array}\right]$$

Thus, the components of geomagnetic vector in a horizontal direction are:

(4) $$\{\begin{array}{l} m_{z}\cos\phi \sin\theta +m_{y}\sin\phi \sin\theta +m_{x}\cos\theta =M_{N}\cos\psi_{m}\\ m_{z}\sin\phi -m_{y}\cos\phi =M_{N}\sin\psi_{m} \end{array}$$

Then, the yaw angle can be expressed as:

(5) $$\psi_{m}=\arctan\left(\frac{M_{y}}{M_{x}}\right)=\arctan\left(-\frac{m_{y}\cos\phi -m_{z}\sin\phi }{m_{x}\cos\theta +m_{y}\sin\theta \sin\phi +m_{z}\sin\theta \cos\phi }\right)$$

In fact, the geomagnetic North Pole and the geographical North Pole do not coincide, and the difference between them is one magnetic declination angle α (calculated by IGRF or WMM geomagnetic model according to the longitude and latitude height of the observation point). Therefore, the real local yaw angle of the carrier obtained based on magnetometer is:

(6) $$\psi_{magn}=\psi_{m}+\alpha$$

#### 2.3.2. Support Attitude Estimation Based on Accelerometer

The accelerometer output is the sensitive carrier acceleration, including the acceleration of the support attitude sensing system and the gravitational acceleration. For support attitude angle computation with an accelerometer, the relationship between the gravitational acceleration ${\left[\begin{array}{ccc} 0 & 0 & g \end{array}\right]}^{T}$ in frame n and the gravitational acceleration $a_{b}={\left[\begin{array}{ccc} a_{x} & a_{y} & a_{z} \end{array}\right]}^{T}$ measured by the accelerometer in frame b could be expressed as:

(7) $$\left[\begin{array}{c} a_{x}\\ a_{y}\\ a_{z} \end{array}\right]=C_{n}^{b}\left[\begin{array}{c} 0\\ 0\\ g \end{array}\right]\left[\begin{array}{c} -g\sin\theta \\ g\cos\theta \sin\phi \\ g\cos\theta \cos\phi \end{array}\right]$$

According to the acceleration output of Equation (7), the roll and pitch angles obtained based on accelerometer can be calculated as follows:

(8) $$\{\begin{array}{l} \theta =-\arctan\left(\frac{a_{x}}{\sqrt{a_{y}^{2}+a_{z}^{2}}}\right)\\ \phi =\arctan\left(\frac{a_{y}}{a_{z}}\right) \end{array}$$

#### 2.3.3. Support Attitude Estimation Based on Gyroscope

The gyroscope output is the sensitive carrier angular acceleration. In this paper, quaternion is applied to calculate the support attitude of hydraulic support. Quaternion has the advantage of faster calculation speed, smooth interpolation, effectively avoiding the universal lock problem and small storage space. Quaternion is a vector composed of four elements, and matrix expression and normalized quaternion of that are defined as follows:

(9) $$q={\left[\begin{array}{cccc} q_{0} & q_{1} & q_{2} & q_{3} \end{array}\right]}^{T},q_{0}^{2}+q_{1}^{2}+q_{2}^{2}+q_{3}^{2}=1$$

Due to the movement of the carrier, *q* is constantly updated. The updated dynamic model based on quaternion using the angular velocity of the gyroscope is expressed as:

(10) $$\dot{q}=\frac{1}{2}q⊗\omega^{b}$$

where $\omega^{b}=\left[\begin{array}{ccc} \omega_{x} & \omega_{y} & \omega_{z} \end{array}\right]$ is the angular velocity and measured based on the gyroscope.

Then, the matrix form of Equation (10) is as follows:

(11) $$\left[\begin{array}{c} {\dot{q}}_{0}\\ {\dot{q}}_{1}\\ {\dot{q}}_{2}\\ {\dot{q}}_{3} \end{array}\right]=\frac{1}{2}\left[\begin{array}{cccc} 0 & -\omega_{x} & -\omega_{y} & -\omega_{z}\\ \omega_{x} & 0 & \omega_{z} & -\omega_{y}\\ \omega_{y} & -\omega_{z} & 0 & \omega_{x}\\ \omega_{z} & \omega_{y} & -\omega_{x} & 0 \end{array}\right]\left[\begin{array}{c} q_{0}\\ q_{1}\\ q_{2}\\ q_{3} \end{array}\right]$$

The Picard successive approximation method of quaternion differential equation is used to solve Equation (11), and the discrete form is as follows:

(12) $$q\left(t_{k}\right)=\left[\cos\left(\frac{\Delta \alpha }{2}\right)I+\sin\left(\frac{\Delta \alpha }{2}\right)\frac{\Delta \Omega }{\Delta \alpha }\right]q\left(t_{k-1}\right)$$

where Δα and ΔΩ involve the integration of angular velocity $\omega^{b}$ in the *k*th sampling period, *k* = 1, 2,… n, $\Delta \alpha =\sqrt{\alpha_{x}^{2}+\alpha_{y}^{2}+\alpha_{z}^{2}}$ is the angular increment of the gyroscope in the [*t_k-1_*,*t_k_*] sampling interval, $\alpha_{i}=\int_{t_{k-1}}^{t_{k}}\omega_{i}dt,i=x,y,z$, and $\Delta \Omega =\int_{t_{k-1}}^{t_{k}}\left[\begin{array}{cccc} 0 & -\omega_{x} & -\omega_{y} & -\omega_{z}\\ \omega_{x} & 0 & \omega_{z} & -\omega_{y}\\ \omega_{y} & -\omega_{z} & 0 & \omega_{x}\\ \omega_{z} & \omega_{y} & -\omega_{x} & 0 \end{array}\right]dt$, *dt* is the sampling interval.

The coordinate transformation matrix expressed by direction cosine, Euler angle and quaternion from carrier coordinate system to the NED coordinate system is written as:

(13) $$\begin{array}{l} C_{b}^{n}=\left[\begin{array}{ccc} C_{11} & C_{12} & C_{13}\\ C_{21} & C_{22} & C_{23}\\ C_{31} & C_{32} & C_{33} \end{array}\right]\\ =\left[\begin{array}{ccc} \cos\theta \cos\psi & \sin\phi \sin\theta \cos\psi -\cos\phi \sin\psi & \cos\phi \sin\theta \cos\psi +\sin\phi \sin\psi \\ \cos\theta \sin\psi & \sin\phi \sin\theta \sin\psi +\cos\phi \cos\psi & \cos\phi \sin\theta \sin\psi -\sin\phi \cos\psi \\ -\sin\theta & \sin\phi \cos\theta & \cos\phi \cos\theta \end{array}\right]\\ =\left[\begin{array}{ccc} q_{1}^{2}-q_{2}^{2}-q_{3}^{2}+q_{0}^{2} & 2\left(q_{1}q_{2}-q_{0}q_{3}\right) & 2\left(q_{0}q_{2}+q_{1}q_{3}\right)\\ 2\left(q_{0}q_{3}+q_{1}q_{2}\right) & -q_{1}^{2}+q_{2}^{2}-q_{3}^{2}+q_{0}^{2} & 2\left(q_{2}q_{3}-q_{0}q_{1}\right)\\ 2\left(q_{1}q_{3}-q_{0}q_{2}\right) & 2\left(q_{0}q_{1}+q_{2}q_{3}\right) & -q_{1}^{2}-q_{2}^{2}+q_{3}^{2}+q_{0}^{2} \end{array}\right] \end{array}$$

Therefore, the support attitude angle can be expressed in the form of Euler angle represented by direction cosine, and is written as:

(14) $$\{\begin{array}{l} \phi =\arctan\frac{C_{32}}{C_{33}}\\ \theta =\arctan\frac{-C_{31}}{\sqrt{C_{32}^{2}+C_{33}^{2}}}\\ \psi =\arctan\frac{C_{21}}{C_{11}} \end{array}$$

The carrier yaw angle in form of Euler angle represented by quaternion is written as:

(15) $$\psi_{gyro}=\arctan\left(\frac{2\left(q_{1}q_{2}+q_{0}q_{3}\right)}{q_{0}^{2}+q_{1}^{2}-q_{2}^{2}-q_{3}^{2}}\right)$$

## 3. The Optimized Quaternion-Based Unscented Kalman Filter

In this section, we propose an unscented Kalman filter based on quaternion for estimating the support attitude of hydraulic support. In order to improve the performance of the support attitude estimation algorithm, the gradient descent algorithm is used for tuning the key parameter of the unscented Kalman filter, i.e., process noise covariance Q. The whole procedure of accurate estimation of support attitude using the gradient descent algorithm-optimized quaternion-based unscented Kalman filter (GD-UKF) is also presented.

### 3.1. Gradient Descent Algorithm

Gradient descent algorithm with the advantages of simple implementation is a common method to solve unconstrained optimization problems. The basic idea of the gradient descent is to select an appropriate initial value $x^{\left(0\right)}$, update the value of x iteratively, and minimize the objective function until it converges. The input of gradient descent is the objective function f(x), the gradient function g(x)=∇f(x) and the calculation accuracy ε; the output is the minimum point $x^{*}$ of f(x). The process of the gradient descent can be summarized in detail as follows:

Step 1.1: Initial key parameters $x^{\left(0\right)}$ and *k*.

Step 1.2: Calculate the objective function $f\left(x^{\left(k\right)}\right)$.

Step 1.3: Calculate the gradient $g_{k}=g(x^{\left(k\right)})$. If $\left\|g_{k}\right\|<\varepsilon$, stop iteration and let $x^{*}=x^{\left(k\right)}$. Otherwise, let $p_{k}=-g(x^{\left(k\right)})$ and then find $\lambda_{k}$ for equation $f\left(x^{\left(k\right)}+\lambda_{k}p_{k}\right)=\underset{\lambda \geq 0}{\min}f(x^{\left(k\right)}+\lambda p_{k})$.

Step 1.4: Set the variable $x^{\left(k+1\right)}=x^{\left(k\right)}+\lambda_{k}p_{k}$ and update the function $f\left(x^{\left(k+1\right)}\right)$. If the expression $\left\|f(x^{\left(k+1\right)})-f(x^{\left(k\right)})\right\|<\varepsilon$ or $\left\|x^{\left(k+1\right)}-x^{\left(k\right)}\right\|<\varepsilon$, stop iteration and let $x^{*}=x^{\left(k\right)}$. Otherwise, let *k* = *k* + 1 and go back to Step 1.3 to find another better minimum.

When the objective function is a convex function, the solution of gradient descent is the global optimal solution.

### 3.2. Unscented Transformation

For unscented Kalman filter in a nonlinear system, unscented transformation (UT) is the core technique for propagating mean and covariance based on Cholesky decomposition, and can effectively approximate mean and covariance changes of the random variables when it undergoes a nonlinear transformation, including cross-correlation between state and measurement. The basic principle of UT can be considered in this way. We assume that the mean and covariance of a random variable *X* with n dimensions are $\bar{x}$ and *P*, respectively, and mean $\bar{x}$ and covariance *P* propagate through a nonlinear function y=f(x). In order to calculate the statistics of the variable y, 2n + 1 sigma points $X^{\left(i\right)}$ and corresponding weights $W^{\left(i\right)}$ are formed and calculated as follows:

(1) Calculate 2n + 1 sigma points
  (16) $$\{\begin{array}{l} X^{\left(0\right)}=\bar{x},i=0\\ X^{\left(i\right)}=\bar{x}+{\left(\sqrt{\left(n+\lambda \right)P}\right)}_{i},i=1∼n\\ X^{\left(i\right)}=\bar{x}-{\left(\sqrt{\left(n+\lambda \right)P}\right)}_{i},i=n+1∼2n \end{array}$$
  where ${\left(\sqrt{p}\right)}^{T}\left(\sqrt{p}\right)=p$, ${\left(\sqrt{p}\right)}_{i}$ is the *i-*th column of the matrix of the square root.
(2) Calculate the corresponding weight of sigma point
  (17) $$\{\begin{array}{l} W_{m}^{\left(0\right)}=\frac{\lambda }{n+\lambda }\\ W_{c}^{\left(0\right)}=\frac{\lambda }{n+\lambda }+\left(1-\alpha^{2}+\beta \right)\\ W_{m}^{\left(i\right)}=W_{c}^{\left(i\right)}=\frac{\lambda }{2\left(n+\lambda \right)},i=1∼2n \end{array}$$
  where $\lambda =\alpha^{2}\left(n+\kappa \right)-n$ is the scaling factor for reducing total prediction error; α is set to a small positive value to control the distribution of sigma points; κ is the parameter to be selected to ensure a positive semidefinite and is usually set to 0; and β is a nonnegative weight coefficient and is set for 2 for Gaussian distribution in this paper. The subscript *m* and *c* represent covariance and mean, respectively.

### 3.3. Unscented Kalman Filter Design

Generally, the dynamic system of the unscented Kalman filter can be described in two system equations: firstly, state equation; and secondly, observation equation. Considering the discrete-time nonlinear dynamic model of support attitude estimation of hydraulic support, the model state variable is $x={\left[\begin{array}{cccc} q_{0} & q_{1} & q_{2} & q_{3} \end{array}\right]}^{T}$, which is a vector composed of four elements of quaternion; and the model observation variable is $z={\left[\begin{array}{ccc} \phi & \theta & \psi \end{array}\right]}^{T}$, which is a vector composed of attitude angles. The state variable is unknown and is constantly updated according to the gyroscope; the observation variable is known and measured by the accelerometer and magnetometer. The details of the mathematical model are described with the following steps.

(1) State equation
  The state prediction is based on the previous optimal estimation, and the discrete-time nonlinear dynamic state equation of unscented Kalman filter is shown as:
  (18) $$x_{k}=A_{k-1}x_{k-1}+w_{k-1}$$
  where $x_{k}$ and $x_{k-1}$ are the prior estimation at time *k* and the posterior estimation at time *k* − 1, respectively; $w_{k}$ is the process noise with covariance Q and simplified as independent Gaussian white noise. $A_{k}$ is the state transition matrix and can be obtained as:
  (19) $$A=\cos\left(\frac{\Delta \alpha }{2}\right)I+\sin\left(\frac{\Delta \alpha }{2}\right)\frac{\Delta \Omega }{\Delta \alpha }$$
(2) Observation equation
  The correction state is the essential step for refining measurement estimation. The observation equation is expressed as
  (20) $$z_{k}=H\left[x_{k}\right]+v_{k}$$
  where $v_{k}$ is the independent Gaussian measurement noise with noise covariance R. $H_{k}\left[x_{k}\right]$ is the output function and can be calculated as follows:
  (21) $$H\left[x\right]=\left[\begin{array}{c} \arctan\left(2\left(q_{2}q_{3}+q_{0}q_{1}\right)/\left(q_{0}^{2}-q_{1}^{2}-q_{2}^{2}+q_{3}^{2}\right)\right)\\ \arcsin\left(2\left(q_{0}q_{2}-q_{1}q_{3}\right)\right)\\ \arctan\left(2\left(q_{1}q_{2}+q_{0}q_{3}\right)/\left(q_{1}^{2}-q_{2}^{2}-q_{3}^{2}+q_{0}^{2}\right)\right) \end{array}\right]$$

### 3.4. Noise Covariance of Process and Observation

The observation noise covariance and process noise covariance are the two key parameters of unscented Kalman filter, which sometimes leads to large support attitude estimation errors. However, it is essential to optimize the noise covariance of the process and observation to minimize the support attitude estimation errors of hydraulic support.

(1) Process noise covariance

In the process of hydraulic support moving and canopy supporting in the automatic coal mining working face, the uneven floor could cause random acceleration of the hydraulic support movement. Meanwhile the coupling effect of roof rock and hydraulic support can produce random impact on the hydraulic support in the moving process and static state, and the left and right adjacent hydraulic support could also produce impact vibration on hydraulic support. Therefore, the coupling effect between hydraulic support and the surrounding environment could lead to random vibration and acceleration on the support attitude sensing system in the static and moving process of hydraulic support, which leads to difficulty in determining the process covariance Q.

We assume that $\omega_{x}={\bar{\omega }}_{x}+w_{x}$, $\omega_{y}={\bar{\omega }}_{y}+w_{y}$, $\omega_{z}={\bar{\omega }}_{z}+w_{z}$ where ${\bar{\omega }}_{x},{\bar{\omega }}_{y},{\bar{\omega }}_{z}$ are the mean of $\omega_{x},\omega_{y},\omega_{z}$; $w_{x},w_{y},w_{z}$ are the process deviations of the support attitude sensing system caused by the disturbance of coupling interaction between the hydraulic support and the surrounding environment. $\sigma_{\omega_{x}}^{2}=E\left(w_{x}\cdot w_{x}^{T}\right)$, $\sigma_{\omega_{y}}^{2}=E\left(w_{y}\cdot w_{y}^{T}\right)$ and $\sigma_{\omega_{z}}^{2}=E\left(w_{z}\cdot w_{z}^{T}\right)$ are the variance of $w_{x},w_{y},w_{z}$. Thus, Equation (11) can be rewritten as:

(22) $$\begin{array}{l} \left[\begin{array}{c} {\dot{q}}_{0}\\ {\dot{q}}_{1}\\ {\dot{q}}_{2}\\ {\dot{q}}_{3} \end{array}\right]=\frac{1}{2}\left[\begin{array}{cccc} 0 & -\left({\bar{\omega }}_{x}+w_{x}\right) & -\left({\bar{\omega }}_{y}+w_{y}\right) & -\left({\bar{\omega }}_{z}+w_{z}\right)\\ \left({\bar{\omega }}_{x}+w_{x}\right) & 0 & \left({\bar{\omega }}_{z}+w_{z}\right) & -\left({\bar{\omega }}_{y}+w_{y}\right)\\ \left({\bar{\omega }}_{y}+w_{y}\right) & -\left({\bar{\omega }}_{z}+w_{z}\right) & 0 & \left({\bar{\omega }}_{x}+w_{x}\right)\\ \left({\bar{\omega }}_{z}+w_{z}\right) & \left({\bar{\omega }}_{y}+w_{y}\right) & -\left({\bar{\omega }}_{x}+w_{x}\right) & 0 \end{array}\right]\left[\begin{array}{c} q_{0}\\ q_{1}\\ q_{2}\\ q_{3} \end{array}\right]\\ =\frac{1}{2}\left[\begin{array}{cccc} 0 & -{\bar{\omega }}_{x} & -{\bar{\omega }}_{y} & -{\bar{\omega }}_{z}\\ {\bar{\omega }}_{x} & 0 & {\bar{\omega }}_{z} & -{\bar{\omega }}_{y}\\ {\bar{\omega }}_{y} & -{\bar{\omega }}_{z} & 0 & {\bar{\omega }}_{x}\\ {\bar{\omega }}_{z} & {\bar{\omega }}_{y} & -{\bar{\omega }}_{x} & 0 \end{array}\right]\left[\begin{array}{c} q_{0}\\ q_{1}\\ q_{2}\\ q_{3} \end{array}\right]+\frac{1}{2}\left[\begin{array}{ccc} -q_{1} & -q_{2} & -q_{3}\\ q_{0} & -q_{3} & q_{2}\\ q_{3} & q_{0} & -q_{1}\\ -q_{2} & q_{1} & q_{0} \end{array}\right]\left[\begin{array}{c} w_{x}\\ w_{y}\\ w_{z} \end{array}\right] \end{array}$$

The second term on the right of Equation (22) can be considered as the process noise $w=\frac{1}{2}\left[\begin{array}{ccc} -q_{1} & -q_{2} & -q_{3}\\ q_{0} & -q_{3} & q_{2}\\ q_{3} & q_{0} & -q_{1}\\ -q_{2} & q_{1} & q_{0} \end{array}\right]\left[\begin{array}{c} w_{x}\\ w_{y}\\ w_{z} \end{array}\right]$ and the process noise covariance Q is:

(23) $$Q=E\left(w\cdot w^{T}\right)=\frac{1}{4}\left[\begin{array}{lll} -q_{1} & -q_{2} & -q_{3}\\ q_{0} & -q_{3} & q_{2}\\ q_{3} & q_{0} & -q_{1}\\ -q_{2} & q_{1} & q_{0} \end{array}\right]\left[\begin{array}{ccc} \sigma_{\omega_{x}}^{2} & 0 & 0\\ 0 & \sigma_{\omega_{y}}^{2} & 0\\ 0 & 0 & \sigma_{\omega_{z}}^{2} \end{array}\right]{\left[\begin{array}{lll} -q_{1} & -q_{2} & -q_{3}\\ q_{0} & -q_{3} & q_{2}\\ q_{3} & q_{0} & -q_{1}\\ -q_{2} & q_{1} & q_{0} \end{array}\right]}^{T}$$

However, it can be determined from Equation (23) that the optimal Q of UKF can be obtained by optimizing variance $\sigma_{\omega_{x}}^{2}$, $\sigma_{\omega_{y}}^{2}$ and $\sigma_{\omega_{z}}^{2}$ based on the optimization algorithm.

(2) Observation noise covariance

The observation noise covariance R is determined by the measurement process and related to the characteristics of the measuring instrument, which can be obtained through long-term probability statistics of sensor measurement data. In this paper, the observation noise covariance R is determined by the measurement deviation of accelerometer and magnetometer.

We assume that $v_{a_{x}}$, $v_{a_{y}}$ and $v_{a_{z}}$ are the observation deviation of accelerometer three-axis output $a_{x}$, $a_{y}$ and $a_{z}$. Then, the first-order Taylor series expansion of trigonometric function is applied to Equation (8), and the deviation of the roll and pitch angles measured by the accelerometer can be derived as

(24) $$\left[\begin{array}{c} v_{\theta }\\ v_{\phi } \end{array}\right]=\left[\begin{array}{ccc} -\frac{\sqrt{a_{y}^{2}+a_{z}^{2}}}{a_{x}^{2}+a_{y}^{2}+a_{z}^{2}} & \frac{a_{x}a_{y}}{\left(a_{x}^{2}+a_{y}^{2}+a_{z}^{2}\right)\sqrt{a_{y}^{2}+a_{z}^{2}}} & \frac{a_{x}a_{z}}{\left(a_{x}^{2}+a_{y}^{2}+a_{z}^{2}\right)\sqrt{a_{y}^{2}+a_{z}^{2}}}\\ 0 & \frac{a_{z}}{a_{y}^{2}+a_{z}^{2}} & -\frac{a_{y}}{a_{y}^{2}+a_{z}^{2}} \end{array}\right]\left[\begin{array}{c} v_{a_{x}}\\ v_{a_{y}}\\ v_{a_{z}} \end{array}\right]=\Phi_{\theta \phi }\left[\begin{array}{c} v_{a_{x}}\\ v_{a_{y}}\\ v_{a_{z}} \end{array}\right]$$

Through Equation (24), the covariance of pitch angle and roll angle based on the accelerometer is as follows:

(25) $$\sigma_{\theta \phi }^{2}=E\left({\left[\begin{array}{cc} v_{\theta } & v_{\phi } \end{array}\right]}^{T}\cdot \left[\begin{array}{cc} v_{\theta } & v_{\phi } \end{array}\right]\right)=\Phi_{\theta \phi }\left[\begin{array}{ccc} \sigma_{a_{x}}^{2} & 0 & 0\\ 0 & \sigma_{a_{y}}^{2} & 0\\ 0 & 0 & \sigma_{a_{z}}^{2} \end{array}\right]\Phi_{\theta \phi }^{T}$$

where $\sigma_{a_{x}}^{2}=E\left(v_{a_{x}}\cdot v_{a_{x}}^{T}\right)$, $\sigma_{a_{y}}^{2}=E\left(v_{a_{y}}\cdot v_{a_{y}}^{T}\right)$ and $\sigma_{a_{z}}^{2}=E\left(v_{a_{z}}\cdot v_{a_{z}}^{T}\right)$ is the variance of accelerometer three-axis output $a_{x}$, and $a_{z}$.

Similarly, through Equations (3) and (5) in the first-order Taylor series expansion of trigonometric function, the deviation of yaw angle measured by magnetometer can be derived as

(26) $$\begin{array}{l} v_{\psi_{magn}}=\left[\begin{array}{cc} -\frac{M_{y}}{M_{x}^{2}+M_{y}^{2}} & \frac{M_{x}}{M_{x}^{2}+M_{y}^{2}} \end{array}\right]\left[\begin{array}{c} M_{x}\\ M_{y} \end{array}\right]\\ =\left[\begin{array}{cc} -\frac{M_{y}}{M_{x}^{2}+M_{y}^{2}} & \frac{M_{x}}{M_{x}^{2}+M_{y}^{2}} \end{array}\right]\left[\begin{array}{ccc} \cos\theta & \sin\theta \sin\phi & \sin\theta \cos\phi \\ 0 & -\cos\phi & \sin\phi \end{array}\right]\left[\begin{array}{c} v_{m_{x}}\\ v_{m_{y}}\\ v_{m_{z}} \end{array}\right]=\Phi_{\psi }\left[\begin{array}{c} v_{m_{x}}\\ v_{m_{y}}\\ v_{m_{z}} \end{array}\right] \end{array}$$

where $v_{m_{x}}$, $v_{m_{y}}$ and $v_{m_{z}}$ are the deviation of magnetometer output $m_{x}$, $m_{y}$ and $m_{z}$.

Through Equation (26), the covariance of yaw angle based on magnetometer is as follows:

(27) $$\sigma_{\psi }=E\left(v_{\psi_{magn}}\cdot v_{\psi_{magn}}^{T}\right)=\Phi_{\psi }\left[\begin{array}{ccc} \sigma_{m_{x}}^{2} & 0 & 0\\ 0 & \sigma_{m_{y}}^{2} & 0\\ 0 & 0 & \sigma_{m_{z}}^{2} \end{array}\right]\Phi_{\psi }^{T}$$

where $\sigma_{m_{x}}^{2}=E\left(v_{m_{x}}\cdot v_{m_{x}}^{T}\right)$, $\sigma_{m_{y}}^{2}=E\left(v_{m_{y}}\cdot v_{m_{y}}^{T}\right)$ and $\sigma_{m_{z}}^{2}=E\left(v_{m_{z}}\cdot v_{m_{z}}^{T}\right)$ are the variances of the magnetometer output $m_{x}$, $m_{y}$ and $m_{z}$, respectively.

Finally, it can be determined from Equations (25) and (27) that the observation noise covariance R is:

(28) $$R=\left[\begin{array}{cc} \sigma_{\theta \phi }^{2} & 0\\ 0 & \sigma_{\psi }^{2} \end{array}\right]$$

### 3.5. Unscented Kalman Filter Based on Gradient Descent

The UKF with Kalman linear filtering framework is not the traditional method of linearizing nonlinear functions, which uses unscented transformation to propagate mean and covariance. Compared with the extended Kalman filter, the UKF without deriving Jacobian matrix can make the state variable approximate the probability density distribution of nonlinear function and is more robust and accurate. However, the process noise covariance Q affects the filtering performance to a certain extent, and gradient descent can be used to improve the unscented Kalman filter. The flow of gradient descent algorithm-optimized quaternion-based unscented Kalman filter (GD-UKF) can be elaborated as below:

Step 2.1: The key parameters of the initial setup are dimension (n = 4), initial value ${\hat{x}}_{0}=E\left(x_{0}\right)$, covariance initial value $P_{0}=E\left(\left(x_{0}-{\hat{x}}_{0}\right){\left(x_{0}-{\hat{x}}_{0}\right)}^{T}\right)$ of random variable. Through Equations (16) and (17), a set of sigma points and the corresponding weights are computed by: i=1,2,⋅⋅⋅,2n+1

(29) $$X_{k-1}^{\left(i\right)}=\left[\begin{array}{ccc} {\hat{x}}_{k-1} & {\hat{x}}_{k-1}+\sqrt{\left(n+\lambda \right)P_{k-1}} & {\hat{x}}_{k-1}-\sqrt{\left(n+\lambda \right)P_{k-1}} \end{array}\right]$$

Step 2.2: The predicted value and covariance matrix of system state variable calculated through the state Equation (18) based on the sigma points obtained in Step 2.1 are expressed as:

(30) $$\begin{array}{l} X_{k}^{\left(i\right)}=A_{k-1}X_{k-1}^{\left(i\right)}\\ {\hat{x}}_{k}^{-}=\sum_{i=0}^{2n}W_{m}^{\left(i\right)}X_{k}^{\left(i\right)}\\ P_{k}^{-}=\sum_{i=0}^{2n}W_{c}^{\left(i\right)}\left[X_{k}^{\left(i\right)}-{\hat{x}}_{k}^{-}\right]{\left[X_{k}^{\left(i\right)}-{\hat{x}}_{k}^{-}\right]}^{T}+Q \end{array}$$

Step 2.3: Through using UT again for the predicted value in Step 2.2, the new sigma points are calculated as follows: i=1,2,⋅⋅⋅,2n+1

(31) $$X_{k}^{\left(i\right)}=\left[\begin{array}{ccc} {\hat{x}}_{k}^{-} & {\hat{x}}_{k}^{-}+\sqrt{\left(n+\lambda \right)P_{k}^{-}} & {\hat{x}}_{k}^{-}-\sqrt{\left(n+\lambda \right)P_{k}^{-}} \end{array}\right]$$

Step 2.4: The predicted value and covariance matrix of observation variable calculated through the observation in Equation (20) based on the new sigma points obtained in Step 2.3 are expressed as follows:i=1,2,⋅⋅⋅,2n+1

(32) $$\begin{array}{l} Z_{k}^{\left(i\right)}=H\left[X_{k}^{\left(i\right)}\right]\\ {\hat{z}}_{k}^{-}=\sum_{i=0}^{2n}W_{m}^{\left(i\right)}Z_{k}^{\left(i\right)}\\ P_{z_{k}z_{k}}=\sum_{i=0}^{2n}W_{c}^{\left(i\right)}\left[Z_{k}^{\left(i\right)}-{\hat{z}}_{k}^{-}\right]{\left[Z_{k}^{\left(i\right)}-{\hat{z}}_{k}^{-}\right]}^{T}+R\\ P_{x_{k}z_{k}}=\sum_{i=0}^{2n}W_{c}^{\left(i\right)}\left[X_{k}^{\left(i\right)}-{\hat{z}}_{k}^{-}\right]{\left[Z_{k}^{\left(i\right)}-{\hat{z}}_{k}^{-}\right]}^{T} \end{array}$$

Step 2.5: The unscented Kalman filter gain matrix *K* is calculated as:

(33) $$K_{k}=P_{x_{k}z_{k}}P_{z_{k}z_{k}}^{-1}$$

Step 2.6: The state update and covariance update of the unscented Kalman filter system are calculated as:

(34) $$\begin{array}{l} {\hat{x}}_{k}={\hat{x}}_{k}^{-}+K_{k}\left[z_{k}-{\hat{z}}_{k}^{-}\right]\\ P_{k}=P_{k}^{-}-K_{k}P_{z_{k}z_{k}}K_{k}^{T} \end{array}$$

**Figure 4:**
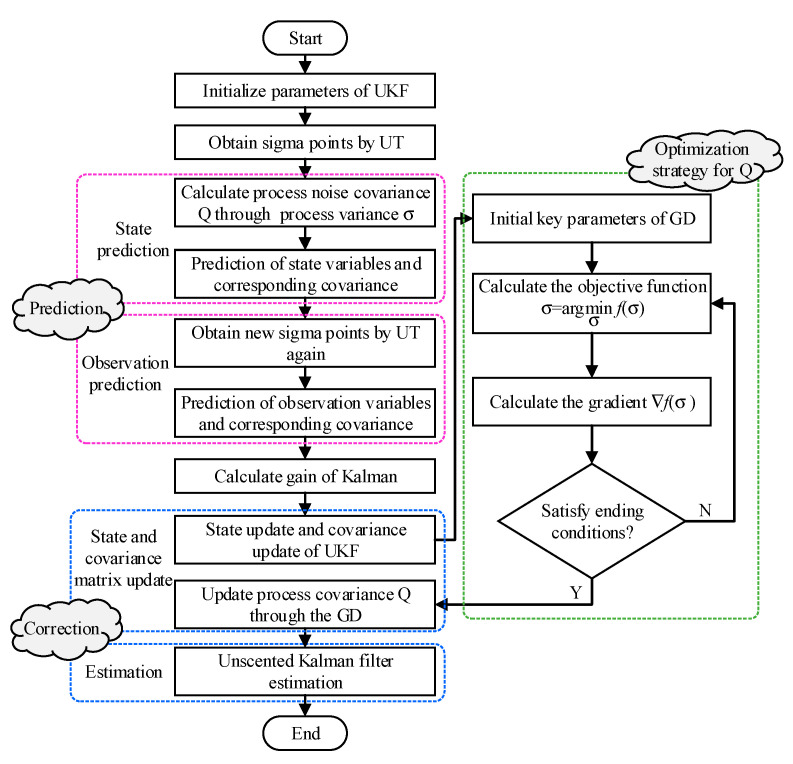
The flowchart of the proposed gradient descent algorithm-optimized quaternion-based unscented Kalman filter.

Step 2.7: The process noise covariance Q is updated by calculating and updating process variance $\sigma ={\left[\begin{array}{ccc} \sigma_{\omega_{x}}^{2} & \sigma_{\omega_{y}}^{2} & \sigma_{\omega_{z}}^{2} \end{array}\right]}^{T}$ of the support attitude sensing system based on gradient descent. And the objective function of gradient descent is

(35) $$\begin{array}{c} f\left(\sigma \right)=L(m_{b},a_{b},\omega^{b},\sigma )=RMSE\left(x,{\hat{x}}_{k}\right)=\sqrt{E\left[\left(x-{\hat{x}}_{k}\right)\cdot {\left(x-{\hat{x}}_{k}\right)}^{T}\right]}\\ \sigma =\underset{\sigma }{\arg\;\min}f(\sigma ) \end{array}$$

where L(⋅) is the loss function; RMSE(⋅) is the root mean square error of estimation results; *x* is the true state value of the support attitude sensing system. The flowchart of the proposed GD-UKF is presented in Figure 4.

## 4. Experiments and Analysis

In this section, in order to evaluate measurement accuracy and performance of the designed support attitude sensing system based on GD-UKF, the static test and dynamic test experiment are carried out on the two-axis experiment platform. Then, the experimental results are contrasted with the Dutch XSENS high-precision attitude measurement system to prove the out-performing and practicability of the designed system.

### 4.1. Experiment on Two-Axis Turntable

**Figure 5:**
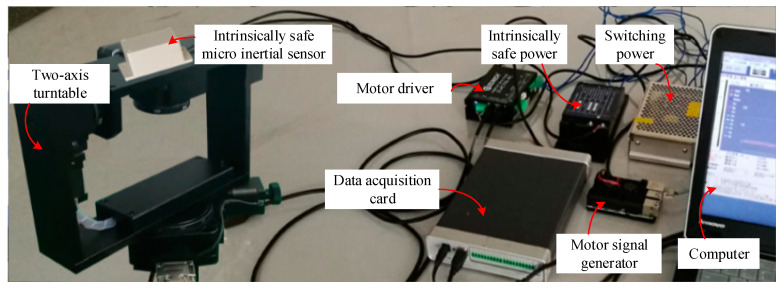
The experimental platform for testing the support attitude sensing system.

The two-axis turntable experimental platform with U-frame structure is a high-precision electric precision machinery and is illustrated in Figure 5. The experiment platform is used for static test with an accuracy of 0.05° and dynamic test with an accuracy of 0.1°. The intrinsically safe micro inertial sensor is mounted on the precision electric rotary table.

In the actual work of hydraulic support in the automatic coal mining working face, the hydraulic supports are closely arranged to support the surrounding rock. According to the coupling relationship between the hydraulic support and surrounding rock, the hydraulic support can appear yaw, pitch and tilt in the movement process, and the allowable range of the canopy pitch is ±15°. Additionally, the roll angle of hydraulic support, that is, the inclination of the coal mining working face, is nonnegative and the maximum value is 40°. The coupling relationship between adjacent hydraulic supports limits the allowable ranges of hydraulic support yaw to ±10°. Therefore, in the experiment setup, the test ranges of pitch, roll and yaw of the experimental platform are set to ±15°, 0° to 40° and ±10°, respectively, which is consistent with the actual working state of hydraulic support. Meanwhile, the attitude angle estimation performance of the support attitude sensing system based on Kalman filter, unscented Kalman filter and the proposed gradient descent algorithm-optimized quaternion-based unscented Kalman filter are tested and compared, respectively. The dynamic test of pitch angle is set from −15° to +15° in 0 s to 200 s, and the angle is maintained at 15° for static test in 200 s to 500 s. The test experiment of roll angle is designed as a dynamic test from 0° to 40° in 0 s to 250 s and static test at 40° in 250 s to 500 s. The dynamic test from −10° to 10° in 0 s to 250 s and the static test at 10° in 250 s to 500 s of yaw angle are set.

### 4.2. Result Analysis

**Figure 6:**
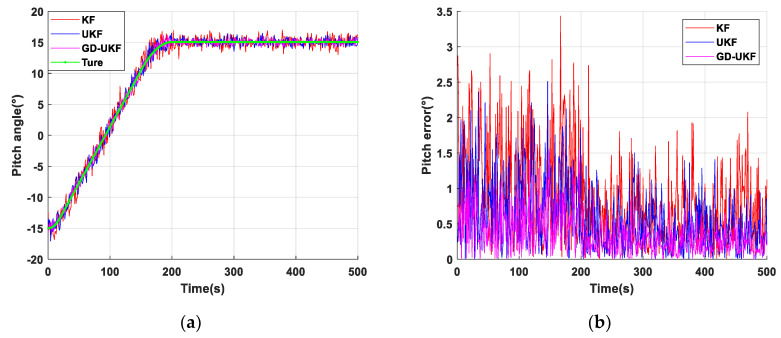
Pitch angle test of support attitude sensing system: (**a**) pitch angle estimation based on three methods; (**b**) pitch error of three methods.

**Figure 7:**
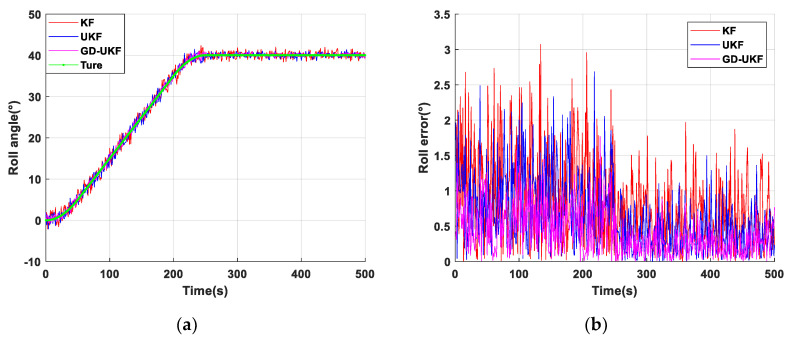
Roll angle test of support attitude sensing system: (**a**) roll angle estimation based on three methods; (**b**) roll error of three methods.

**Figure 8:**
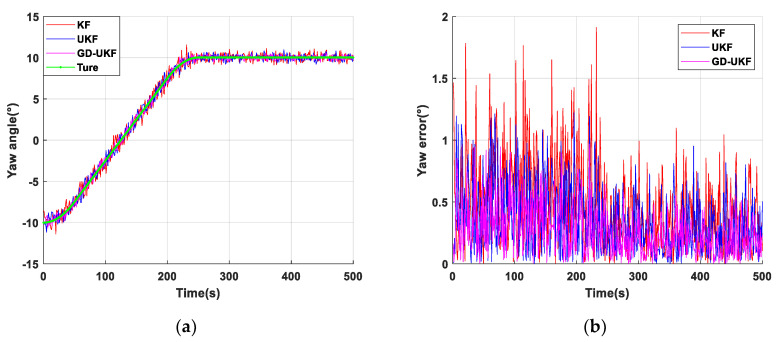
Yaw angle test of support attitude sensing system: (**a**) yaw angle estimation based on three methods; (**b**) yaw error of three methods.

The experimental results of the support attitude sensing system-based KF, UKF and GD-UKF on the two-axis experimental platform are shown in Figure 6, Figure 7 and Figure 8, in which Figure 6, Figure 7 and Figure 8a show three attitude angles measured by the three methods, and the attitude calculation results of the three methods are consistent with the variational trend set by the experimental platform. In this paper, the root mean square error is used to evaluate the estimation accuracy of the algorithms, and the estimation errors of the three attitude angles are shown in Figure 6b, Figure 7 and Figure 8. The estimation errors (shown in the red curve) of pitch angle and roll angle based on Kalman filter are relatively large, and the maximum errors are greater than 3°, as shown in Figure 6b and Figure 7b. Compared with the Kalman filter, the unscented Kalman filter improves the estimation accuracy to a certain extent, the blue curve is closer to the green curve than the red curve, but the estimation errors with maximum error greater than 2.4° are still relatively large. The reason for this is that the covariance Q, the key parameter of the algorithm, is set to the empirical value. In Figure 6 and Figure 7, the estimation value (the pink curve) based on the proposed GD-UKF, is rather close to the theoretical value (the green curve), the dynamic and static estimation errors are less than 1° and 0.5°, respectively. The reason is that Q is tuned by gradient descent, and the variances $\sigma_{\omega_{x}}^{2}$, $\sigma_{\omega_{y}}^{2}$ and $\sigma_{\omega_{z}}^{2}$ in the optimized Q matrix are infinitely close to the true process noise covariance value caused by the inherent deviation of the experimental platform, as shown in Table 1, which greatly improves the estimation performance of UKF. Therefore, the estimation errors of pitch angle and roll angle based on GD-UKF are distinctly smaller than those of UKF and KF methods, and the accuracies of dynamic estimation and static estimation are better than 1° and 0.5°, respectively.

As shown in Figure 8a, the fluctuation of yaw angle estimation results of three methods are stable. The maximum estimation errors based on KF and UKF are 1.8° and 1.3°, respectively, as shown in Figure 8b, the average estimation error of yaw angle of GD-UKF is 0.2° to 0.4° smaller than that of the other two algorithms, which is not obviously demonstrated to have the superior estimation performance of the proposed algorithm. The reason is that there is no magnetic field interference such as high-power equipment in the laboratory, and the estimations of yaw angle by the three methods are almost the same in the environment without magnetic noise interference. For the estimation performance of yaw angle based on GD-UKF, an industrial application needs to be operated in the automatic coal mining working face to validate the superiority and practicability of the proposed algorithm in yaw angle estimation.

The accuracy of our support attitude sensing system, with the static measurement accuracy better than 0.5° and dynamic measurement accuracy better than 1°, is lower than that of MTi-630 AHRS based on industrial-grade MEMS sensor from XSENS [43], but the support attitude sensing system is much cheaper than MTi-630 AHRS and its size is also smaller, which can meet the support accuracy requirements of hydraulic support. Moreover, the support attitude sensing system with 200 mW power consumption has lower power consumption than MTi-630 AHRS with 345 mW power consumption and especially meets the technical requirements of an intrinsically safe system [44], which can be directly applied to the automatic coal mining working face in explosive atmospheres. Considering the support accuracy requirement, the total price and power consumption of sensors and intrinsically safe technology requirements in the automatic coal mining working face, we argue that the designed support attitude sensing system is more suitable for the special application scenarios with a large demand for the number of sensors and complex environment. Therefore, it can be directly applied to measure the support attitude of hydraulic support canopy, and is also more suitable for the coal mine special environment navigation applications of other mobile equipment like mine inspection robots, support robots, driving robots, and so on.

## 5. Industrial Experiment and Application

**Figure 9:**
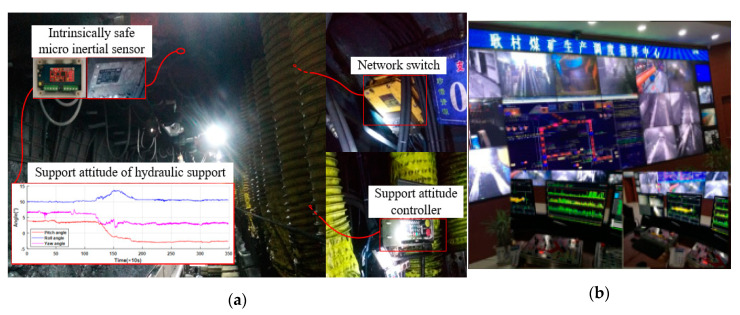
Industrial application of the support attitude sensing system: (**a**) application environment; (**b**) remote monitoring center.

In this section, our support attitude sensing system based on the proposed GD-UKF is applied in the automatic coal mining working face to test practical performance, as shown in Figure 9. The industrial experiment and application were tested at the 13230 coal mining working face of Gengcun Mine of Yima Coal Industrial Group Co., Ltd. (Yima, China). The intrinsically safe micro inertial sensor was installed on the canopy of hydraulic support to measure support attitude of hydraulic support. The attitude angle information was transmitted to the remote monitoring center by the network switch, and then the support attitude of hydraulic support was adjusted by the support attitude controller to satisfy the support requirements of coal mining.

**Figure 10:**
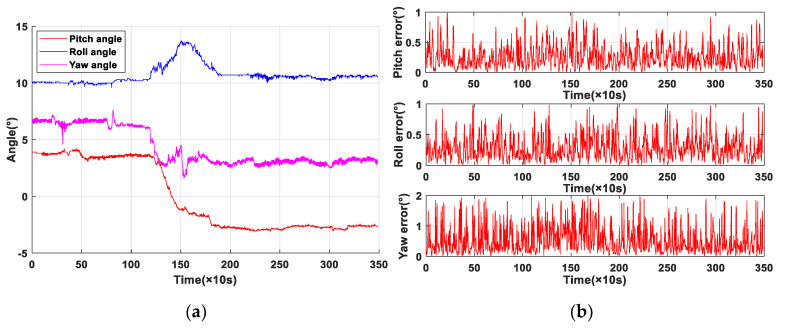
Industrial application results of support attitude sensing system-based GD-UKF: (**a**) attitude angle estimation; (**b**) estimation error.

**Figure 11:**
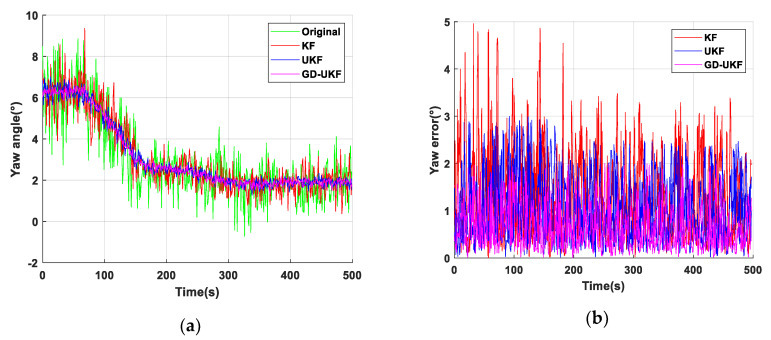
Estimation results of yaw angle tested in industrial experiment: (**a**) yaw angle estimation based on three methods; (**b**) estimation error of three methods.

The initial pitch angle of the hydraulic support canopy was 3.5°, then the angle was adjusted to −2.6° and remained stationary. In this process, the roll angle fluctuated and finally returned to the initial angle of 10° which was the inclination of the 13,230 coal mining working face, and the yaw angle was also changed from 6.9° to 3.9°. The industrial application results using the support attitude sensing system are shown in Figure 10. The pitch and roll estimation errors of support sensing system base GD-UDF were less than 1°, which could meet the requirements of the automatic coal mining working face. In order to verify the feasibility and superiority of the proposed algorithm for magnetic disturbances filtering, an industrial experiment of yaw angle was carried out, and the test results were shown in Figure 11. However, we could obviously find out that the yaw angle had a larger variation range than the other two angles and the yaw estimation error was relatively large, as shown in Figure 11a, the measurement error of original data was about 5° and that of yaw angle based on GD-UKF was less than 2°. The reason is that the high-power equipment, such as a shearer in the automatic coal mining working face, can generate a certain complex external magnetic field interference, which has a great influence on the yaw estimation of hydraulic support and is difficult to eliminate.

The industrial application has verified that the support attitude estimation of hydraulic support with our support attitude sensing system using gradient descent algorithm-optimized quaternion-based unscented Kalman filter for attitude solution has high measurement accuracy, but the movement conditions of the automatic coal mining working face are complex, especially the magnetic disturbance. Therefore, the experiments for improving the yaw angle estimation algorithm need to be carried out under more complicated conditions.

## 6. Conclusions and Future Work

In order to tackle the problem of support attitude measurement of hydraulic support, this paper proposes a support attitude sensing system with the feature of intrinsic safety based on the low-cost MEMS-IMU and STM32MP157. The proposed gradient descent algorithm-optimized quaternion-based unscented Kalman filter makes full use of the characteristics of complementation of gyroscope, accelerometer and magnetometer to improve the accuracy of attitude estimation. In the proposed method, the gradient descent algorithm is employed to tune the process noise covariance for optimizing the state forecasting estimation. To verify the attitude estimation performance of the designed system and the proposed approach, an experiment was carried out and some analysis and comparisons were conducted. The experiment analysis results show that the support attitude sensing system based on the proposed approach has accurate attitude estimation performance with a dynamic measurement precision better than 1° and a static measurement precision better than 0.5°. Finally, the industrial experiment and application for estimating support attitude of hydraulic support in the automatic coal mining working face were performed to validate the practicability and feasibility of the designed support attitude sensing system.

However, the error elimination of support attitude sensing system is still a challenge, especially eliminating the estimation error of yaw angle in the environment of complex magnetic disturbance. In future studies, the authors will focus on algorithm research on error elimination in the complex magnetic perturbation environment. These studies may include updating the covariance of the algorithm in combination with other potential interference factors affecting support attitude estimation to further improve the estimation accuracy. In addition, the tuning parameter intelligent algorithm based on the maximum likelihood estimation of mathematic statistics is also worth further research, and the covariance is approximately solved based on the statistical model trained by machine learning with existing data.

## Figures and Tables

**Table 1:** Process noise covariance.

	Inherent Covariance of Experimental Platform	Covariance Optimized by Gradient Descent
Static test	$\left[\begin{array}{ccc} 0.0025 & 0 & 0\\ 0 & 0.0025 & 0\\ 0 & 0 & 0.0025 \end{array}\right]$	$\left[\begin{array}{ccc} 0.0046 & 0 & 0\\ 0 & 0.0053 & 0\\ 0 & 0 & 0.0032 \end{array}\right]$
Dynamic test	$\left[\begin{array}{ccc} 0.0100 & 0 & 0\\ 0 & 0.0100 & 0\\ 0 & 0 & 0.0100 \end{array}\right]$	$\left[\begin{array}{ccc} 0.0196 & 0 & 0\\ 0 & 0.0182 & 0\\ 0 & 0 & 0.0134 \end{array}\right]$

## References

[1] Wang G., Liu F., Pang Y., Ren H., Ma Y. (2019). Coal mine intellectualization: The core technology of high quality development. J. China Coal. Soc..

[2] Hu S., Tang C., Yu R., Liu F., Wang X. Intelligent coal mine monitoring system based on the Internet of Things. Proceedings of the 2013 3rd International Conference on Consumer Electronics, Communications and Networks.

[3] Wang J., Huang Z. (2017). The recent technological development of intelligent mining. Engineering.

[4] Guan E., Miao H., Li P., Liu J., Zhao Y. (2019). Dynamic model analysis of hydraulic support. Adv. Mech. Eng..

[5] Gwiazda A. (2014). Design of the roof support with strait-Line mechanism. Adv. Mater. Res..

[6] Wang G., Wang L., Guo Y. (2014). Determining the support capacity based on roof and coal wall control. J. China. Coal. Soc..

[7] Zhang Y., Zhang H., Gao K., Xu W., Zeng Q. (2019). New method and experiment for detecting relative position and posture of the hydraulic support. IEEE Access.

[8] Cao L., Sun S., Zhang Y., Guo H., Zhang Z. (2018). The research on characteristics of hydraulic support advancing control system in coal mining face. Wirel. Pers. Commun..

[9] Yang X., Wang R., Wang H., Yang Y. (2020). A novel method for measuring pose of hydraulic supports relative to inspection robot using LiDAR. Measurement.

[10] Meng Z., Zeng Q., Wan L., Liu P. (2018). Pose adjusting simulation of hydraulic support based on mechanical-electrical-hydraulic coordination. Teh. Vjesn..

[11] Zeng X., Meng G., Zhou J. (2018). Analysis on the pose and dynamic response of hydraulic support under dual impact loads. Int. J. Simul. Model..

[12] Ge X., Xie J., Wang X., Liu Y., Shi H. (2020). A virtual adjustment method and experimental study of the support attitude of hydraulic support groups in propulsion state. Measurement.

[13] Liang M., Fang X., Li S., Wu G., Ma M., Zhang Y. (2019). A fiber Bragg grating tilt sensor for posture monitoring of hydraulic supports in coal mine working face. Measurement.

[14] Pareek S., Manjunath H., Esfahani E.T., Kesavadas T. (2019). MyoTrack: Realtime estimation of subject participation in robotic Rehabilitation using sEMG and IMU. IEEE Access.

[15] Xu Z., Lihua D., Zhong S., Ning L. (2018). Study of the navigation method for a snake robot based on the kinematics model with MEMS IMU. Sensors.

[16] Wen K., Yu K., Li Y., Zhang S., Zhang W. (2020). A New quaternion Kalman Filter based foot-mounted IMU and UWB tightly-coupled method for indoor pedestrian navigation. IEEE Trans. Veh. Technol..

[17] Hu G., Zhang W., Wan H., Li X. (2020). Improving the heading accuracy in indoor pedestrian navigation based on a decision tree and Kalman Filter. Sensors.

[18] Hachaj T., Piekarczyk M. (2019). Evaluation of pattern recognition methods for head gesture-based interface of a virtual reality helmet equipped with a single IMU sensor. Sensors.

[19] Chen Z., Zhu Q., Soh Y.C. (2016). Smartphone inertial sensor-based indoor localization and tracking with iBeacon corrections. IEEE Trans. Ind. Inform..

[20] Zhong M., Guo J., Yang Z. (2016). On real time performance evaluation of the inertial sensors for INS/GPS integrated systems. IEEE Sens. J..

[21] Wu Y., Luo S. (2016). On misalignment between magnetometer and inertial sensors. IEEE Sens. J..

[22] Ko N., Jeong S., Bae Y. (2016). Sine rotation vector method for attitude estimation of an underwater robot. Sensors.

[23] Chung H., Ojeda L., Borenstein J. (2001). Accurate mobile robot dead-reckoning with a precision-calibrated fiber-optic gyroscope. Robot. Autom. IEEE Trans..

[24] Hong S.K., Park S. (2008). Minimal-drift heading measurement using a MEMS gyro for indoor mobile robots. Sensors.

[25] Hamid K.R., Talukder A., Islam A.K.M.E. (2018). Implementation of fuzzy aided Kalman filter for tracking a moving object in two-dimensional. Int. J. Fuzzy Log. Intell. Syst..

[26] Hu P., Xu P., Chen B., Wu Q. (2018). A self-calibration method for the installation errors of rotation axes based on the asynchronous rotation of rotational inertial navigation systems. IEEE Trans. Ind. Electron..

[27] Jiménez A.R., Seco F., Prieto J.C., Guevara J. Indoor pedestrian navigation using an INS/EKF framework for yaw drift reduction and a foot-mounted IMU. Proceedings of the 7th Workshop on Positioning, Navigation and Communication (WPNC 2010).

[28] Wang G., Li N., Zhang Y. (2017). Hybrid consensus sigma point approximation nonlinear filter using statistical linearization. Trans. Inst. Meas. Control.

[29] Zhao J., Mili L. (2018). A robust generalized-maximum likelihood unscented Kalman Filter for power system dynamic state estimation. IEEE J. Sel. Top. Signal Process..

[30] Deng Z., Wang G., Hu Y., Wu D. (2015). Heading estimation for indoor pedestrian navigation using a smartphone in the pocket. Sensors.

[31] Abdelrahman A., Naser E.S. (2013). Low-cost MEMS-based pedestrian navigation technique for GPS-denied areas. J. Sens..

[32] Chopin N., Jacob P.E., Papaspiliopoulos O. (2013). SMC 2: An efficient algorithm for sequential analysis of state space models. J. R. Stat. Soc..

[33] Carvalho C.M., Johannes M.S., Lopes H.F., Polson N.G. (2010). Particle learning and smoothing. Stat. Sci..

[34] Martino L., Elvira V., Camps-Valls G. (2018). Group importance sampling for particle filtering and MCMC. Digit. Signal Process..

[35] Lingo Urteaga M., Bugallo F., Djuric P.M. Sequential Monte Carlo methods under model uncertainty. Proceedings of the IEEE statistical Signal Processing Workshop.

[36] Martino L., Elvira V., Camps-Valls G. Distributed particle metropolis-Hastings schemes. Proceedings of the IEEE Statistical Signal Processing Workshop.

[37] Yang C., Shi W., Chen W. (2017). Comparison of unscented and extended Kalman Filters with application in vehicle navigation. J. Navig..

[38] Yuan X., Yu S., Zhang S., Wang G., Liu S. (2015). Quaternion-based unscented Kalman Filter for accurate indoor heading estimation using wearable multi-sensor system. Sensors.

[39] Zhang L., Sidoti D., Bienkowski A., Pattipati K.R., Kleinman D.L. (2020). On the identification of noise covariances and adaptive Kalman Filtering: A new look at a 50 year-old problem. IEEE Access.

[40] Zhang X., Li P., Tu R., Lu X., Ge M., Schuh H. (2020). Automatic calibration of process noise matrix and measurement noise covariance for multi-GNSS precise point positioning. Mathematics.

[41] Poddar S., Kottath R., Kumar V., Kumar A. (2016). Adaptive sliding Kalman filter using nonparametric change point detection. Measurement.

[42] Murray R., Swenson B., Kar S. (2019). Revisiting normalized gradient descent: Fast evasion of saddle points. IEEE Trans. Autom. Control.

[43] MTi-630 AHRS. https://www.xsens.com/products/mti-600-series.

[44] Explosive Atmospheres (2010). Part 18: Intrinsically Safe System.

